# An Unanswered Question about Diabetes Mellitus

**DOI:** 10.5812/nms.11200

**Published:** 2013-06-27

**Authors:** Maryam Houshmand

**Affiliations:** 1 Department of Medical-Surgical Nursing, Faculty of Nursing and Midwifery, Kashan University of Medical Sciences, Kashan, IR Iran

**Keywords:** Diabetes Mellitus, Question, Unanswered

Dear Editor,

Diabetes Mellitus is so prevalent all around the world. As known, Diabetes is accounted among the high morbid and mortal diseases. As a nurse I had a question in mind, I wanted to know "what is the best or assuring criterion to judge on the improvement in diabetic patients?". I asked some physicians, some of them emphasized that fasting blood sugar (FBS) and postprandial blood sugar are the important items in evaluating the patients' conditions. Others insisted on evaluating based on the glycosylated hemoglobin (HbA1c) test results. While searching on the internet with the keyword diabetes mellitus, I found five articles in your journal's archive including the term "diabetes" (1-5). Three of them were included the term diabetes in the title or keywords: two qualitative researches and a randomized controlled trial (1-3). I read those articles and I found them so great and professionally written. What made me to write this letter to you was that in the studies conducted to evaluate the effects of different complementary medicines on diabetic patients, I found no assuring criterion to evaluate the patients' improvement. Most of the worldwide published studies evaluated the patients' improvement through laboratory tests. For example one of the published studies in your journal evaluated the patients' improvement based on their blood sugar levels in the morning (fasting blood sugar) and in the afternoon (blood sugar at 5 pm) (1). In a clinical review published in 2009 reviewing the clinical evidence supporting complementary and alternative medicine interventions for improving glycemic control in type 2 diabetic patients, HbA1c and fasting blood glucose (FBG) levels were reported as the criteria for evaluating the effectiveness of specific therapies (6). Based on The National Diabetes Education Program, the intermediate outcomes in glucose control are achieving/maintaining healthy weight, self-monitoring of blood glucose (SMBG) levels in the target range, HbA1c levels below 7%, blood pressure below 130/80 mm Hg and Low-density lipoprotein cholesterol (LDL) levels below 100 mg/dL (7). American diabetes association introduced patient SMBG and HbA1c to assess the effectiveness of the management plan on glycemic control and also considered medical history, physical examination, laboratory evaluation, and referrals as the components of comprehensive diabetes evaluation. The authors of this standard recommended that patients should do SMBG 6-8 times daily, and perform HbA1c test 2-4 times a year or even more if needed (8). However, there is the fact that the amount of blood glucose depends on food composition, portion size (the amount of consumed food) and timing of meals and snacks (9). Also, HbA1c test is affected by erythrocyte turnover and hemoglobin variants. In addition, sometimes the laboratory test results do not match the clinical conditions of the patients (8). Both of the blood glucose (fasting or postprandial) and HbA1c are affected by different conditions and may represent false interpretations of patients' health, however these two items are being used frequently to judge patients' conditions worldwide. As a conclusion, I found each item for evaluating the effectiveness of the management plan on glycemic control faces with limitations. Finally this question is still in my mind that "what is the best or assuring criterion to judge the improvement in diabetic patients?". It seems that researches are needed to find the precise answer to this question.
